# Effect of Co-Inoculation with *Saccharomyces cerevisiae* and Lactic Acid Bacteria on the Content of Propan-2-ol, Acetaldehyde and Weak Acids in Fermented Distillery Mashes

**DOI:** 10.3390/ijms20071659

**Published:** 2019-04-03

**Authors:** Katarzyna Pielech-Przybylska, Maria Balcerek, Grzegorz Ciepielowski, Barbara Pacholczyk-Sienicka, Łukasz Albrecht, Urszula Dziekońska-Kubczak, Radosław Bonikowski, Piotr Patelski

**Affiliations:** 1 Department of Spirit and Yeast Technology, Institute of Fermentation Technology and Microbiology, Faculty of Biotechnology and Food Sciences, Lodz University of Technology, Wolczanska 171/173, 90-924 Lodz, Poland; maria.balcerek@p.lodz.pl (M.B.); urszula.dziekonska-kubczak@p.lodz.pl (U.D.-K.); piotr.patelski@p.lodz.pl (P.P.); 2 Institute of Organic Chemistry, Faculty of Chemistry, Lodz University of Technology, Zeromskiego 116, 90-924 Lodz, Poland; grzegorz.ciepielowski@p.lodz.pl (G.C.); barbara.pacholczyk@p.lodz.pl (B.P.-S.); lukasz.albrecht@p.lodz.pl (Ł.A.); 3 Institute of General Food Chemistry, Faculty of Biotechnology and Food Sciences, Lodz University of Technology, Stefanowskiego 4/10, 90-924 Lodz, Poland; radoslaw.bonikowski@p.lodz.pl

**Keywords:** lactic acid bacteria (LAB), acetone, propan-2-ol, thermal-pressure method of starch liberation

## Abstract

The qualitative and quantitative composition of volatile compounds in fermented distillery mash determines the quality of the obtained distillate of agricultural origin (i.e., raw spirit) and the effectiveness of further purification steps. Propan-2-ol (syn. isopropyl alcohol), due to its low boiling point, is difficult to remove by rectification. Therefore, its synthesis needs to be limited during fermentation by *Saccharomyces cerevisiae* yeast, while at the same time controlling the levels of acetaldehyde and acetic acid, which are likewise known to determine the quality of raw spirit. Lactic acid bacteria (LAB) are a common but undesirable contaminant in distillery mashes. They are responsible for the production of undesirable compounds, which can affect synthesis of propan-2-ol. Some bacteria strains are able to synthesize isopropyl alcohol. This study therefore set out to investigate whether LAB with *S. cerevisiae* yeast are responsible for conversion of acetone to propan-2-ol, as well as the effects of the amount of LAB inoculum and fermentation parameters (pH and temperature) on the content of isopropyl alcohol, acetaldehyde, lactic acid and acetic acid in fermented mashes. The results of NMR and comprehensive two-dimensional gas chromatography coupled with time of flight mass spectrometry (GC × GC-TOF MS) analysis confirmed the ability of the yeast and LAB strains to metabolize acetone via its reduction to isopropyl alcohol. Efficient fermentation of distillery mashes was observed in all tested mashes with an initial LAB count of 3.34–6.34 log cfu/mL, which had no significant effect on the ethanol content. However, changes were observed in the contents of by-products. Lowering the initial pH of the mashes to 4.5, without and with LAB (3.34–4.34 log cfu/mL), resulted in a decrease in propan-2-ol and a concomitant increase in acetaldehyde content, while a higher pH (5.0 and 5.5) increased the content of propan-2-ol and decreased acetaldehyde content. Higher temperature (35 °C) promoted propan-2-ol synthesis and also resulted in increased acetic acid content in the fermented mashes compared to the controls. Moreover, the acetic acid content rose with increases in the initial pH and the initial LAB count.

## 1. Introduction

The basic raw material for the production of pure vodka is ethyl alcohol of agricultural origin (i.e., rectified spirit), which is obtained from a distillate of agricultural origin via the rectification process. An EU regulation [1] specifies the requirements for ethyl alcohol of agricultural origin, while the quality requirements for distillate of agricultural origin are regulated by the individual standards of EU countries [2]. In raw spirit, only the levels of aldehydes (calculated as acetaldehyde) and acids (calculated as acetic acid) are limited [2], while in rectified spirit, the content of higher alcohols, methanol and esters is restricted [1]. The concentration of these compounds significantly decreases during the rectification process. However, some, such as isopropyl alcohol, belonging to the higher alcohols group, cannot be completely removed, due to the similarity between their boiling points and that of ethanol [3]. Vodkas produced in EU countries which are exported to other non-EU countries must fulfil the additional requirements of the foreign recipients, as has been detailed in a previous work [3]. One of these additional restrictions is the level of propanol-2-ol in pure vodkas and ethyl alcohol of agricultural origin [3]. Therefore, the content of isopropyl alcohol should be controlled. However, due to the difficulty of removing this compound in the rectification process, the synthesis of isopropyl alcohol needs to be limited during the process of fermenting distillery mashes.

Propan-2-ol is formed via acetone reduction, which, in turn, can be formed through enzymatic reactions (from pyruvate) [4] or chemical reactions under severe conditions (as a product of Maillard reactions) [5]. The enzyme that catalyzes the reduction of acetone to isopropyl alcohol is alcohol dehydrogenase. Yeast alcohol dehydrogenases are the most active towards acetaldehyde and ethanol [6]. Moreover, yeasts synthesize other alcohols, including minor and major fusel alcohols (C3–C8) [7]. Some bacteria strains are also well-known producers of primary and secondary higher alcohols, including butan-1-ol, butan-2-ol, propan-1-ol and propan-2-ol [4]. In distillery mashes, other microorganisms occur, apart from yeast, particularly lactic acid bacteria (LAB), which are both common and undesirable [8,9,10,11,12]. The acetic and lactic acids produced by LAB are the most common inhibitors of the fermentation process. Both acids have a negative effect on the growth of yeast, although acetic acid has been found to be much more toxic to yeast than lactic acid [13]. Thomas et al. [12] observed a decrease in yeast proliferation and increased loss of yeast cell viability with an acetic acid content of 1.1–2.0 g/L. The presence of acetic and lactic acids is associated with lower external pH (pH_ex_) during the process of ethanol fermentation. Lower pH_ex_ promotes the migration of undissociated acids into yeast cells [13,14]. Due to their dissociation, these weak acids can then cause a decrease in the intracellular pH (pH_in_) of yeast cells. The optimal pH for glycolytic enzymes and alcoholgenic enzymes is close to neutral [4]. Yeast can efficiently regulate its pH_in_ to remain within the optimal range (pH 6–7) across a wide range of pH_ex_ [14,15]. However, the presence of acids disturbs that balance [14]. The negative effect of weak acids can also be enhanced by the temperature and pH of the fermented medium [13,14,16]. Consequently, lower pH_in_ can decrease the activity of enzymes responsible for glucose metabolism and ethanol synthesis in the fermentation process [17]. For example, decreases in the activity of alcohol dehydrogenase affect the accumulation of acetaldehyde, with a concomitant decrease in ethanol content. Such disruption in its activity can also result in changes in the concentration of other aldehydes and some ketones, including acetone, as well as of higher alcohols [6,7]. Moreover, the presence of undesirable microbiota can negatively affect the activity of yeast alcohol dehydrogenase, due to the presence of their metabolites [14].

In our previous studies [18], we observed the presence of acetone in sweet distillery mash prepared using the thermal-pressure method for starch liberation. Propan-2-ol was detected at the end of fermentations carried out with both yeast and lactic acid bacteria. Based on the results, the present research set out to investigate the influence of microorganisms present in distillery mashes and of fermentation conditions on acetone metabolism and isopropyl alcohol synthesis. The impact of these variables on the quality of the raw spirit, i.e., levels of acetaldehyde and acetic acid, was also evaluated. The main aims of the present work were, therefore: (i) to confirm whether the acetone present in sweet mash is metabolized to propan-2-ol by *Saccharomyces cerevisiae* yeast and five strains of lactic acid bacteria (used as a mixed culture); (ii) to examined the effect of the three variables, i.e., initial pH of sweet rye distillery mashes, the fermentation temperature and the initial LAB count, on the propan-2-ol, acetaldehyde and weak acids content in mashes fermented by *S. cerevisiae* yeast.

## 2. Results and Discussion

### 2.1. Synthesis of Propan-2-ol from Acetone-2-^13^C

A number of microorganisms, especially anaerobic bacteria, are able to synthetize isopropyl alcohol [4,19]. This secondary alcohol may be found in distillery mashes [18], as well as in distillates [3]. However, there is nothing in the literature that proves whether acetone, which occurs in sweet mashes as a product of the thermal-pressure treatment of cereal grains, can be a source of isopropyl alcohol synthesis during ethanol fermentation in the presence of yeast and/or bacteria. In our previous research [18], we observed that acetone content decreased during fermentation of mashes using *S. cerevisiae* and LAB separately. The decrease in acetone content was accompanied by the appearance of propan-2-ol in the fermented mashes, perhaps indicating that both yeast and LAB are responsible for the reduction of this ketone to isopropyl alcohol. Given the results of our previous research [18], in the first part of the present study we set out to confirm whether *S. cerevisiae* yeast with five strains of lactic acid bacteria (used as a mixed culture) were able to metabolize acetone to isopropyl alcohol. The lactic acid bacteria strains used in our experiments were selected based on the results of a detailed microbiological analysis of distillery mashes by Broda and Leja [8]. It was decided to use a mixed LAB culture containing four strains of *Lactobacillus* sp. and one strain of *Lactococcus* sp.

**Figure 1:**
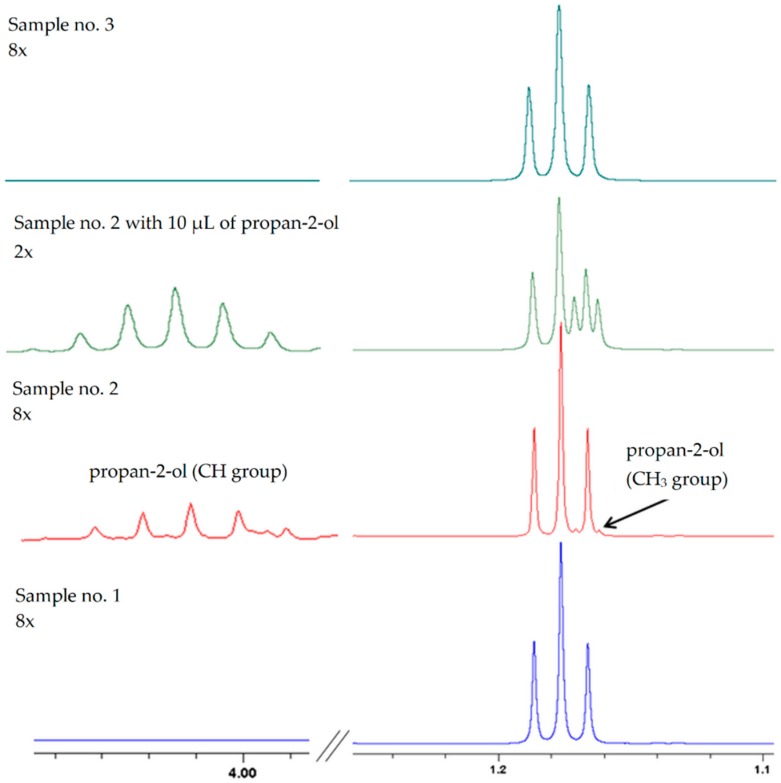
^1^H NMR spectra in the chemical shift region associated with propan-2-ol signals. The methyl group is located at 1.16 ppm, the methine group at 4.01 ppm. (Sample no. 1—distillate obtained from mash inoculated with yeast; Sample no. 2—distillate obtained from mash inoculated with lactic acid bacteria (LAB); Sample no. 3—distillate obtained from mash inoculated with yeast and LAB).

Sweet mash fermentations (initial pH 5.0, at 35 °C) were first performed with the addition of external acetone with isotope-labeled carbon (1 mg of acetone-2-^13^C/L of sweet mash). The total soluble solids and total content of sugars in the sweet mash were 140.8 ± 10.4 k/kg and 128.4 ± 9.6 g glucose/L, respectively. Prior to the fermentations, the sweet mashes were inoculated separately with *S. cerevisiae* yeast (sample no. 1) (initial yeast count 6.45 log cfu/mL), with LAB strains (in a mixed culture) (sample no. 2) (initial LAB count 6.00 log cfu/mL), or co-inoculated both with *S. cerevisiae* yeast (6.45 log cfu/mL) and LAB (6.00 log cfu/mL) (sample no. 3). After fermentation (72 h), ethanol was distilled from the mashes and the distillates were subjected to NMR and comprehensive two-dimensional gas chromatography coupled with time of flight mass spectrometry (GC × GC-TOF MS) analysis. Figure 1 shows the part of the ^1^H NMR spectrum in which signals from propan-2-ol should occur. In the case of sample no. 2 (the red spectrum—distillate obtained from mash inoculated with LAB), signals from the CH_3_ group of ethanol can be seen located at 1.18 ppm. This overlaps with the signal from the methyl group of propan-2-ol at 1.16 ppm. Due to the low concentrations of isopropyl alcohol in all the samples, the signal from the CH group located at 4.01 ppm was enlarged eight times. The presence of propan-2-ol was confirmed on the basis of standard addition.

**Figure 2:**
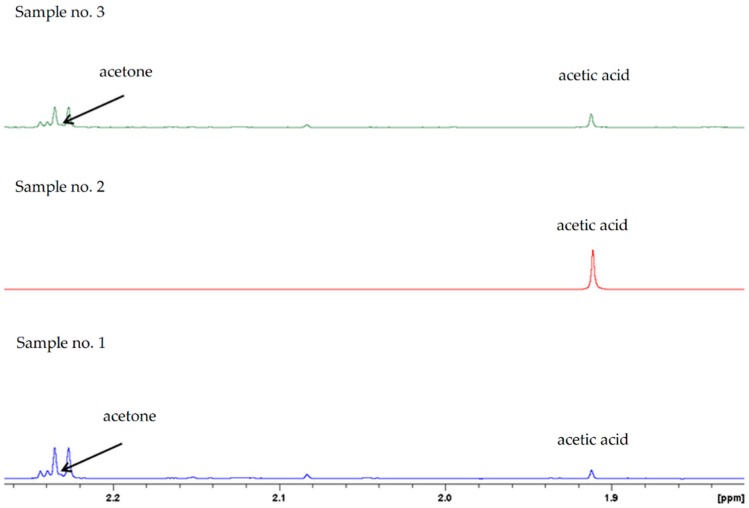
^1^H NMR spectra in the chemical shift region associated with acetone and acetic acid signals. (Sample no. 1—distillate obtained from mash inoculated with yeast; Sample no. 2—distillate obtained from mash inoculated with LAB; Sample no. 3—distillate obtained from mash inoculated with yeast and LAB).

On the green spectrum (sample no. 2), it can be seen that signals from propan-2-ol increased after the addition of 10 μL of the standard. Moreover, in the case of sample no. 2, in which propan-2-ol was detected, no signals from acetone were observed at 2.21 ppm (in the red spectrum) (Figure 2). This suggests that it may be formed from acetone. In the case of sample no. 1 (distillate obtained from mash inoculated with *S. cerevisiae* yeast), a signal was observed from acetone (Figure 2, in the blue spectrum), but not from propan-2-ol (Figure 1, in the blue spectrum). The concentration of propan-2-ol may have been below the level of detection. Analogous results to those for sample no. 1 were obtained for sample no. 3 (distillate obtained from mash inoculated with yeast and mixed culture of LAB) (Figure 1 and Figure 2).

**Figure 3:**
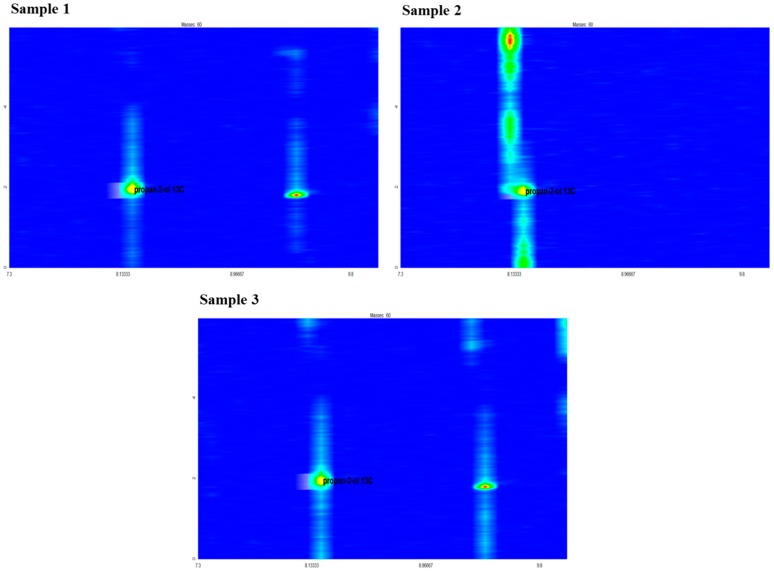
Results of comprehensive two-dimensional gas chromatography coupled with time of flight mass spectrometry (GC × GC-TOF MS) analysis of distillates. (Sample no. 1—distillate from mash inoculated with *Saccharomyces cerevisiae* yeast; Sample no. 2—distillate from mash inoculated with LAB; Sample no. 3—distillate from mash inoculated with *S. cerevisiae* yeast and LAB).

Next, a GC analysis of the distillates was carried out using two-dimensional gas chromatography with time-of-flight mass spectrometry. The results for samples no. 1–3 (Figure 3) confirmed the presence of propan-2-ol-2-^13^C. We concluded that, in the presence of both *S. cerevisiae* yeast and LAB strains (inoculated separately), acetone was converted into isopropyl alcohol (samples no. 1 and 2). Moreover, co-inoculation of sweet mashes with *S. cerevisiae* yeast and LAB resulted in the formation of propan-2-ol-2-^13^C during the fermentation period (sample no. 3). We could not, however, determine in the case of sample no. 3 whether it was the yeast, the bacteria, or both, that was responsible for the metabolism of the acetone to propan-2-ol.

Hasino [20] examined the ability of *Lactobacillus brevis* var. *hofuensis* to produce propan-2-ol via the reduction of acetone. It was confirmed that various microorganisms, including some LAB strains (*Lactobacillus* sp. and *Leuconostoc* sp.), *Bacillus* sp. strains and *Saccharomyces* sp. strains (*S. cerevisiae* and *S. formosensis*) are able to reduce acetone efficiently. However, there is no information in the literature on how the presence of LAB affects the metabolism of acetone by yeast during ethanol fermentation, or whether the LAB are still able to efficiently reduce acetone to propan-2-ol in the presence of yeast.

### 2.2. Effect of Fermentation Parameters and Initial Amount of LAB Inoculation on Isopropyl alcohol, Acetaldehyde, Lactic Acid and Acetic Acid Content in Fermented Mashes

In the second part of the study, we evaluated three variables (initial LAB count, initial pH and temperature) on acetone metabolism and propan-2-ol formation during the fermentation of rye mashes obtained by the thermal-pressure method of starch liberation. To inoculate the sweet mashes, the same microorganisms were used as in the first part of the study. The fermentation temperature (27 and 35 °C), the initial pH (4.5, 5.0 and 5.5) and the initial LAB count in the sweet mash (3.34, 4.34, 5.34 and 6.34 log cfu/mL) were recorded. The initial LAB count used in our study was established based on results reported by Broda and Leja [8] following a microbial analysis of distillery mashes obtained on an industrial scale.

The contents of ethanol, residual sugars, acetic and lactic acids, acetaldehyde and glycerol were also determined, to evaluate the progress of fermentation. We assessed the extent to which the applied variables influenced the final yeast count and LAB in the fermented mash. The results are presented in Table 1 and Table 2. A statistical analysis is given in Table 3.

To better understand and explain the results of chemical analysis, with particular focus on volatile compounds including acetone, propan-2-ol, acetaldehyde and acetic acid, we first assessed the results of microbial analysis of the fermented mashes. All three examined variables were demonstrated to affect the final LAB count, while there were no significant differences in yeast count in any of the analyzed mashes (Table 1). The LAB count in the co-fermented mashes increased in all trials (*p* < 0.05). While assessing the effect of pH and temperature on LAB growth, it was observed that the trials fermented at 35 °C with lower initial pH (4.5) had a lower LAB count, compared to analogous samples fermented at 27 °C. This dependence was observed in trials inoculated with LAB inoculum in the range of 3.34 to 5.34 log cfu/mL. Adamberg et al. [21] evaluated the influence of temperature on the growth of selected *Lactobacillus* strains and showed, inter alia, that the specific growth rate of the *Lactobacillus acidophilus* strain was highest at 41 °C, while that for *Lactobacillus delbrueckii* was highest at 43 °C. In turn, the *Lactococcus lactis* strain reached a maximum growth rate at about 35 °C. Ahmed et al. [22] showed that strains of *L. lactis* and *L. acidophilus* grow efficiently in a temperature range of 37–40 °C. The *Lactobacillus fermentum* strain showed similar growth requirements in studies conducted by Garro et al. [23]. The LAB strains used by us should, therefore, grow efficiently at a higher temperature, but this was not borne out in our studies (Table 1), probably due to mutual interactions between the LAB and yeast. Ethanol and other metabolites that appear during fermentation may also limit LAB growth more efficiently at higher temperatures. The growth of LAB is also affected by pH and the chemical composition of the medium. Most LAB strains grow more slowly at low pH, preferring pH levels in the range of 5–6. Some, such as *L. acidophilus* and *L. delbrueckii*, have the ability to regulate their pH_in_ even in slightly acidic environments [24]. The acids produced by LAB at low pH can cause damage to bacteria cells and loss of viability [25,26]. However, in our research, the LAB count did not affect the final yeast count in the fermented mashes, even in the samples with higher lactic acid content (Table 1 and Table 2). This was probably due to the high natural resistance of *S. cerevisiae* yeast to low pH [26,27].

Statistical analysis (Table 3) showed that temperature had only a slightly effect on the final number of yeast cells. The average number of yeast cells in the fermented samples at 27 °C was 8.17 log cfu/mL, while at 35 °C it was 7.89 log cfu/mL. The remaining variables did not affect the final concentration of yeast cells. Thomas et al. [12] report that when the percentage of yeast cells to bacteria at the beginning of fermentation was larger by 22%–50%, the multiplication of bacteria was effectively inhibited, due to the increased use of nutrients by the yeast as well as by the ethanol present in the medium. Low pH may further enhance these effects. In our research, use of sweet mash with a lower initial pH (4.5) resulted in a lower LAB count in the fermented mashes, compared to the samples with a higher pH (5.0 and 5.5). This was also visible in the concentration of lactic acid, which did not exceed 0.2 g/L in the samples with pH 4.5, but increased in those at pH 5.0 and 5.5. When the yeast and LAB counts were similar (6.34 log cfu/mL), there was an increase in the concentration of lactic acid, to 3.05 and 3.7 g/L in the case of pH 5.0 and to 4.46 and 4.64 g/L at pH 5.5 (Table 1). Narendranath et al. [10] also observed an increase in lactic acid concentrations with increasing levels of bacteria inoculation. In our study, no negative effect on ethanol content or yeast count was observed for LAB in the range of 3.34–6.34 log cfu/mL in fermented mashes (Table 1 and Table 2). However, a higher LAB count may disrupt the course and yield of fermentation [10].

The process of ethanol fermentation is accompanied by the use of sugars as well as the synthesis of glycerol and weak acids, including acetic and lactic acid. The temperature and initial pH affected the content of residual sugars (Table 2 and Table 3). Lower concentrations of sugars were noted in the samples fermented at 35 °C compared to the samples fermented at 27 °C. When the initial pH of the sweet mashes was increased, the concentration of sugars also decreased. The glycerol in distillery mashes originates during yeast metabolism. Among the main environmental factors affecting glycerol content are the sugar content, the temperature and the pH. Higher values for these factors improve the glycerol yield [28]. In our studies, an increase in glycerol content was observed in the mash as the initial pH and temperature increased. The presence of lactic acid bacteria at an initial count of 3.34 and 5.34 log cfu/mL did not affect the glycerol content significantly in comparison to the control samples. Nevertheless, in trials with an initial LAB count of 6.34 log cfu/mL, the glycerol content decreased when the initial pH was increased from 5.0 to 5.5. One of the reasons is the metabolism of glycerol, which involves lactic acid bacteria. Glycerol is reduced to 3-hydroxypropionaldehyde, followed by reduction to 1,3-propanediol. Garai-Ibabe et al. [29] isolated 22 LAB strains from spoiled ciders which degrade glycerol via the glycerol dehydratase pathway.

Lactic acid bacteria use sugars in two main ways: via the homofermentative pathway, forming lactic acid, and by the heterofermentative pathway, forming lactic acid, acetic acid, formic acid and ethanol [24]. In our study, lactic acid was the major acid found in the fermented mashes, in quantities ranging from 0.1 to 4.64 g/L (Table 2). Lactic acid was not detected in the control samples. Acetic acid, in turn, occurred in both non-inoculated samples as well as in those inoculated with LAB. Acetic acid is formed by some lactic acid bacteria strains, but *S. cerevisiae* yeast are also capable of its synthesis [3,30,31]. Moreover, the thermal-pressure method used in distilleries for the treatment of starchy raw materials may be a source of this acid, which is formed via Maillard reactions [5]. In our study, co-inoculation with yeast and LAB (at 35 °C) increased the acetic acid content in the fermented mashes compared to the control samples. Moreover, the acetic acid content rose with increases in the initial pH and the initial LAB count (Table 2 and Table 3). The increase in acetic acid content may cause higher total acidity in raw spirits obtained from distillation, reducing their quality in terms of normative requirements [2].

As can be seen, LAB content of 3.34–6.34 log cfu/mL in mashes fermented by *S. cerevisiae* yeast did not influence the ethanol content. In the first part of the current study, we observed that the investigated strains of yeast and lactic acid bacteria metabolized acetone to propan-2-ol. We then decided to examine the effect of the fermentation parameters (initial pH and temperature), as well as of the initial LAB count, on the propan-2-ol concentration in the fermented mashes. Based on the results and statistical analysis (Table 2 and Table 3), we concluded that the isopropyl alcohol content was affected by all three variables. The content of propan-2-ol in the control samples (without LAB inoculation) ranged from 0.46 to 0.66 mg/L, while the highest values were noted in mashes when the initial pH was set at 5.5. Inoculation with LAB at 3.34 and 4.34 log cfu/mL did not change this dependence. As the initial pH was increased, the concentration of isopropyl alcohol in the analyzed mash samples also rose, while subsequent increases in the LAB inoculation ratio (to 5.34 and 6.34 log cfu/mL) resulted in a lower content of isopropyl alcohol. Temperature also had an impact on propan-2-ol content. Differences were observed in the samples with the highest initial pH (5.5) (Table 2). In the trial fermented at 27 °C, the content of isopropyl alcohol was lower compared to samples fermented at 35 °C.

A reverse relation was observed in the case of acetone. The results for isopropyl alcohol were thereby confirmed by quantitative analysis of the acetone content in the fermented mashes. The highest content of this ketone was determined in fermented mash samples when the initial pH was set at 4.5. The concentration of acetone decreased with increasing initial pH. Comparison of analogous samples (with the same initial pH) further revealed the influence of temperature. In mash fermented at a higher temperature (35 °C), the concentration of acetone was lower compared to mash fermented at 27 °C.

The results of the control trials confirm that the yeast used in our studies metabolize acetone to propan-2-ol. This process was supported by higher initial pH, as well as by higher fermentation temperature (Table 2). Moreover, when used in a mixed culture, the tested LAB strains showed the ability to synthesize isopropyl alcohol (Figure 1, Figure 2 and Figure 3). Nevertheless, the presence of LAB in mashes inoculated with yeast did not result in an increase in the concentration of isopropyl alcohol in comparison to the control samples (inoculated with yeast only). The content of isopropyl alcohol in fact decreased in the mash inoculated with a larger LAB inoculum (5.34 and 6.34 log cfu/mL) (Table 2).

We also evaluated the acetaldehyde concentration, which is considered as an indicator of the quality of raw spirit. Acetaldehyde content increases during ethanol fermentation and reaches its highest values in the early fermentation phase, before decreasing in the later phase [32]. Analysis of the acetaldehyde content revealed certain analogies to the results for acetone and isopropyl alcohol. Increasing the initial pH in the controls contributed to increase the concentration of isopropyl alcohol, while the concentration of acetaldehyde was lower. On the other hand, a higher LAB count at the beginning of fermentation (6.34 log cfu/mL) with a higher initial pH (pH 5.5) contributed to increase the content of acetaldehyde more than two-fold, compared to analogous controls. Lower amounts of LAB (3.34–5.34 log cfu/mL) did not increase the concentration of acetaldehyde, which in some cases even fell compared to the controls. Some bacteria are able to produce both acetic acid and ethanol, without or with the simultaneous production of lactic acid. Therefore, we cannot exclude the possibility that the lower concentration of acetaldehyde in these samples may be due to its reduction by LAB [33]. However, we did not observe a significant increase in the concentration of ethyl alcohol in the samples (Table 2). Another reason could be the synthesis of acetals, including acetaldehyde diethyl acetal. Acidic conditions promote the formation of acetals [34]. Kłosowski and Czupryński [35] observed an increase in the content of acetaldehyde diethyl acetal over successive hours during fermentation of distillery mashes.

Weak acids present in the medium affect the pH_in_ of yeast cells. However, as reported by Pampulha and Loureiro-Dias [14], the pH_in_ of *S. cerevisiae* yeast cells depends on the concentration of the undissociated form of acetic acid, not on the content of total acetic acid present in the medium. Changes to the pH_in_ of *S. cerevisiae* yeast cells may subsequently lead to the inhibition of enzymes responsible for sugar metabolism and the synthesis of fermentation by-products. Narendranath et al. [36] examined both acetic and lactic acids on changes to the pH_in_ of *S. cerevisiae* yeast. They found that the pH_in_ of the strains was not affected by acetic acid at concentrations <0.25% *w*/*v*, whereas lactic acid in concentrations above 0.4% *w*/*v* significantly affected the pH_in_. Pampulha and Loureiro-Dias [14] found that the pH_in_ of yeast cells in control samples (without the presence of acetic acid) remained unchanged, regardless of the pH of the medium (3.5, 4.5, or 5.5), and there was no negative impact on the efficiency of fermentation. In the presence of acetic acid, decreases in both the pH_in_ and the fermentation rate were observed. Moreover, the presence of ethyl alcohol in the medium intensified the negative effect of acetic acid.

In our studies, the lowest final pH of the fermented mashes (3.7 and 3.8) and the highest lactic acid content were observed in samples with a higher initial LAB count (5.34 and 6.34 log cfu/mL). The increasing content of weak acids in the medium over time probably affected the pH_in_ of the yeast cells and the activity of their enzymes. The high initial pH values used in our research probably ensured that the yeast enzymes, including alcohol dehydrogenase, had high activity, hence there was a higher concentration of isopropyl alcohol and a lower acetaldehyde concentration in the control samples (initial pH 5.5). Yeast are able to produce ethanol with high-yields, but only for a brief period in the fermentation process, after which the yield decreases [15]. Therefore, it is important to ensure optimum parameters, including low microbial contamination, for high yeast fermentation activity in the subsequent stages of the fermentation process.

## 3. Materials and Methods

### 3.1. Materials

Rye grains of the Dankowskie Diament variety were used as the starchy raw material for sweet mash.

The following microorganisms were used for sweet mash inoculation:

— dry distillery Ethanol Red yeast (*Saccharomyces cerevisiae*) (Fermentis Division of S.I. Lesaffre, France),
— lactic acid bacteria strains: *Lactobacillus acidophilus* Ł0842, *Lactobacillus delbrueckii* Ł0854, *Lactobacillus casei* Ł0901, *Lactobacillus fermentum* T53 Ł0954 and *Lactococcus lactis* Ł0877, obtained from the Pure Cultures Collection of Industrial Microorganisms at the Institute of Fermentation Technology and Microbiology (ŁOCK 105, Lodz University of Technology, Poland).

### 3.2. Preparation of Lactic Acid Bacteria Inoculum 

The sweet mashes were inoculated with a mixture of five LAB strains. All the bacteria strains were grown separately in 500 mL flat-bottom flasks containing 250 mL of MRS (De Man, Rogosa and Sharpe) broth (Merck, Burlington, MA, USA), under anaerobic growth conditions (30 °C, 48 h). The bacterial cells were then separated from the broth by centrifugation at 23,300× *g* for 15 min at 4 °C and washed twice with sterile saline solution (0.9% *w*/*v* NaCl). Next, all bacteria strains cells were suspended separately in 20 mL of saline solution and the concentration of bacteria cells was estimated using a DEN-1B densitometer (Biosan, Riga, Latvia). The bacterial inoculum was prepared by mixing an appropriate volume of suspensions of all bacteria strains to achieve final cell concentrations of 8 log cfu/mL, with the same number of cells from each bacterial strain. Inoculation with mixed lactic acid bacteria was performed by taking 1 mL of inoculum suspension from subsequent dilutions to achieve final bacteria cell concentrations in the sweet mash samples (100 mL) of approx. 3, 4, 5 and 6 log cfu/mL. For NMR experiments, the sweet mashes (700 mL) were inoculated with LAB inoculum to achieve a final cell concentration of 6 log cfu/mL.

### 3.3. Preparation of Yeast Inoculum

Prior to fermentation, yeast slurry was prepared by suspending an appropriate amount of dried distillery yeast in distilled water (0.5 g/L of sweet mash, yeast count 6.45 ± 0.32 log cfu/mL of sweet mash). Hydration of the yeast cells and disinfection of the yeast slurry was performed by acidification using sulfuric (VI) acid solution (the final pH of the yeast slurry was set at 2.5). The yeast slurry was kept at room temperature for 15 min to eliminate weaker yeast cells and undesirable bacterial cells. It was then added to the sweet mash samples.

### 3.4. Sweet Mash Processing

All experiments were based on rye sweet mash. The rye sweet mash was processed using the thermal-pressure method, with two-fold circulation to avoid high acetone content formation via Maillard reactions. A tapered cylindrical steamer [37] was filled with 17.5 L of water brought to boiling point using superheated steam, then 5 kg of rye grain was added. The steamer was closed and the pressure inside the steamer was slowly increased to 0.15 MPa. This temperature was maintained for 10 min. The content of the steamer was circulated by opening a vent valve, thereby lowering the pressure to 0.1 MPa. Next, the pressure was increased to 0.35 MPa and maintained at this level for 10 min, followed by a second circulation (the pressure was decreased to 0.25 MPa). Finally, the pressure was increased to 0.4 MPa and maintained for 25 min.

In the second part of the study, the rye grains were treated using only one circulation (at 0.15 MPa), following which the pressure was increased to 0.4 MPa and maintained for 40 min.

On completion of the thermal-pressure treatment process, the mashing process was performed according to a procedure described previously [37], using enzymes of microbial origin.

Prior to fermentation, the sweet mash was supplemented with diammonium phosphate (0.2 g/L).

### 3.5. Fermentation

Before inoculation with yeast and/or LAB, the initial pH of the fermentation trails (prepared in triplicate) was set at 4.5, 5.0 or 5.5 using sulfuric (VI) acid solution. After inoculation of the sweet mash, the fermentations were continued for 72 h, in two thermostatic rooms, with the temperature set to 27 or 35 °C. Before and after fermentation, mash samples were collected for analysis.

#### 3.5.1. Synthesis of Propan-2-ol from Acetone-2-^13^C

Fermentation of the sweet mash samples (pH 5.0) was carried using radiolabeled acetone (acetone-2-^13^C, Sigma-Aldrich) at a final concentration of 1 mg/L. In a 2 L glass flask, 700 mL of sweet mash was inoculated with yeast slurry or bacterial inoculum separately, or with yeast slurry and bacterial inoculum simultaneously.

To protect the mashes against the development of undesirable microorganisms, solutions of antibiotics (penicillin G sodium salt, 100,000 U/L mash and streptomycin sulfate salt 0.1 g/L mash) or nystatin (0.06 g/L), respectively, were added through a sterile filter to the sweet mash samples inoculated with yeast or LAB. The glass flasks were closed with an airlock filled with glycerin. All the mash samples were fermented at 35 °C, for 72 h. When the fermentation process was complete, samples of the fermented mashes were collected for distillation prior to NMR and GC × GC-TOF MS analysis.

#### 3.5.2. Effect of LAB Count in Sweet Mash on Propan-2-ol Content 

The pH of the sweet mash samples was set at 4.5, 5.0 or 5.5. Fermentations (in triplicate) were carried without the addition of external acetone. In a 250 mL glass flask, 100 mL of sweet mash was inoculated with yeast slurry and the bacterial inoculum. The control was sweet mash inoculated with yeast only. To protect the mashes against the development of bacteria, antibiotics (penicillin G sodium salt, 100 000 U/L mash and streptomycin sulfate salt 0.1 g/L mash) were added to the control sample solutions. The glass flasks were closed with a glycerin-filled air lock. The mash samples were fermented at 27 or 35 °C, for 72 h. After 72 h, the fermented mashes were collected for chemical and microbial analysis.

### 3.6. Distillation

The distillation process was carried out according to a procedure described elsewhere [38].

### 3.7. Analysis of Sweet and Fermented Mash 

#### 3.7.1. Chemical Composition 

The chemical composition of the sweet and fermented mashes was evaluated using the dinitrosalicylic method [39] (total sugars before fermentation and residual sugars after completion of fermentation, preceded by acid hydrolysis) and HPLC analysis [37] (acetic acid, lactic acid, glycerol and ethanol content). The content of total dissolved solids in the sweet mash was assessed using a hydrometer, according to a method described elsewhere, with the results presented in g/kg [38].

#### 3.7.2. Microbial Analysis of Mashes

The sweet and fermented mashes were analyzed for yeast (DRBC medium, Merck, Burlington, MA, USA; growth at 25 °C for five days) (ISO 21527-1:2008) [40] and LAB (MRS medium with nystatin, Merck, Burlington, MA, USA; anaerobic growth at 30 °C for 72 h) (ISO 4833:2004) [41]. Prior to analysis, the samples were prepared according to the ISO 6887 method (ISO 6887-1:1999) [42]. The limit of detection was 10 cfu/mL. The results of microbial analysis were presented as log cfu/mL.

#### 3.7.3. HS-GC-MS Analysis of Sweet and Fermented Mashes

Qualitative and quantitative analyses of the content of acetone, propan-2-ol and acetaldehyde were performed according to a method described previously [18]. The results of GC-MS analysis were expressed in mg/L of mash. All analyses were performed in triplicate.

### 3.8. Analysis of Distillate 

To evaluate the ability of the microorganisms (i.e., *S. cerevisiae* yeast and LAB strains) to metabolize acetone (i.e., acetone 2-^13^C) to propan-2-ol (i.e., propan-2-ol-2-^13^C) during fermentation, the distillates obtained from the fermented mashes were analyzed using NMR and gas chromatography (GC×GC-TOF MS).

#### 3.8.1. NMR Analysis 

All samples for NMR measurements were prepared in the same way, as follows: 50 µL of the analyzed distillate was added to 600 µL of deuterium oxide containing 0.02 mM TSP (sodium-3′-trimethylsilylpropionate-2,2,3,3-d4). The samples (650 µL) were transferred immediately to 5 mm NMR tubes for NMR measurements. All spectra were acquired using a Bruker Avance II Plus 16.4 T spectrometer (Bruker BioSpin, Germany) operating at 1H frequency (700.44 MHz). The instrument was equipped with a 5 mm Z-gradient broadband decoupling inverse probe. All experiments were conducted at 300 K. Standard proton spectra with water presaturation (zgcppr pulse program, Bruker) were acquired with a calibrated 90° pulse for 64 scans, collecting 32 K data points over a spectral width of 11,439 Hz. The repetition time of 10.4 s, including a relaxation delay of 8 s, was calculated as 7T1 of the longest relaxation time needed to ensure complete magnetization recovery. An exponential line broadening of 0.05 Hz was applied to the raw data prior to Fourier transformation. The TSP peak at 0 ppm was used as a chemical shift standard. All spectral regions were individually corrected using a fifth-order baseline function. Quantitative analysis was performed for the spectral regions from 0 to 6 ppm.

#### 3.8.2. GC×GC-TOF MS Analysis

To confirm the presence of propan-2-ol-2-^13^C, solid phase microextraction (SPME) combined with comprehensive two-dimensional gas chromatography coupled with mass spectrometry (GC×GC-TOF MS) was used. Analyses were performed using a Pegasus 4D apparatus (LECO, St. Joseph, MI, USA). Separation was carried out using an Rtx-BAC1 as a GC oven column (length 30 m, internal diameter 0.32 mm, film thickness 1.8 µm; Restek Corporation, Bellefonte, USA) and BPX-50 as a secondary oven column (length 2 m, internal diameter 0.1 mm, film thickness 0.1 µm; Trajan Scientific, Ringwood, Australia). The GC oven temperature program was from 50 °C (for 7 min) to 150 °C at 4 °C/min. The two-stage consumable free modulator was cooled to −80 °C. Temperature offsets relative to the GC oven were as follows: secondary oven +5°, modulator +20°. Modulation period 6 s, hot pulse time 1.8 s, cooling time between stages 1.2 s. Helium was used as the carrier gas at a flow rate of 1.5 mL/min. Mass spectra were collected using a time-of-flight mass spectrometer (TOF MS). The settings for TOF MS were as follows: ion source temperature 200 °C, ionization energy 70 eV, acquisition voltage 200 V above the optimized voltage, scan range 33–650 at 150 spectra/s. The mass spectrum of propan-2-ol-2-^13^C is: MS (70 eV) *m*/*z* (%) 61(1) [M+], 46(100), 44(28), 40(12), 42(11), 45(8), 43(7), 60(3). The data were analyzed using LECO ChromaTOF software (v. 4.50.8.0).

### 3.9. Statistical Analysis

Statistical calculations were performed using STATISTICA 6.0 software (Tibco Software, Palo Alto, CA, USA). Three-way ANOVA was used (at a 0.05 significance level) to evaluate the differences between the tested sweet mash samples, using as variables the amount of LAB inoculation, the fermentation temperature and the initial pH of the sweet mash. When statistical differences were found (*p* < 0.05), the means were compared using the post hoc Duncan test (at a 0.05 significance level).

## 4. Conclusions

The initial pH of the distillery mashes in agricultural distilleries is set at a high level, optimal for yeast activity, in the range of 5–5.5. As confirmed by this study, this practice guarantees high ethanol yield and low concentrations of acetaldehyde. Undesirable microbiota, mainly lactic acid bacteria (LAB), are a common presence in mashes. Given the high nutritional and environmental requirements of LAB for optimal growth, it is advisable to reduce both their initial count in the sweet mash and its initial pH, in order to limit their development. This study has shown that lowering the initial pH of sweet mash to 4.5 has a positive effect on limiting LAB growth and decreasing the concentration of their metabolites (lactic and acetic acids), without significantly affecting yeast growth or ethanol content. A lower content of isopropyl alcohol was also observed, probably due to the lower activity of alcohol dehydrogenase. However, there was an increase in acetaldehyde content, the main quality indicator for raw spirit. Temperature also had an effect of the content of this compound, as did the LAB count at the beginning of the fermentation process. A higher initial LAB count (over 4 log cfu/mL) and a high initial pH (5.5) promoted LAB growth, leading to higher concentrations of weak acids and higher acetaldehyde content. We conclude that the studied LAB strains are able to metabolize acetone and synthesize isopropyl alcohol efficiently. However, in the presence of yeast as the predominant microorganism, the LAB strains were unable to effectively reduce acetone to 2-propanol.

## Figures and Tables

**Table 1:** Microbial analysis of fermented mashes.

LAB Count in Sweet Mash log cfu/mL	Temperature (°C)	Initial pH	Fermented Mash	
			LAB Count log cfu/mL	Yeast * Count log cfu/mL
Mean ± SD			Mean ± SD	Mean ± SD
Control sample (no inoculation with LAB)	27	4.5	<1.00 _a_	8.18 ± 0.38 _a_
		5.0	<1.00 _a_	8.21 ± 0.38 _a_
		5.5	<1.00 _a_	8.14 ± 0.38 _a_
	35	4.5	<1.00 _a_	8.01 ± 0.37 _a_
		5.0	<1.00 _a_	7.93 ± 0.37 _a_
		5.5	<1.00 _a_	8.00 ± 0.37 _a_
3.34 ± 0.18	27	4.5	5.64 ± 0.30 _c_	8.17 ± 0.43 _a_
		5.0	6.36 ± 0.34 _de_	8.21 ± 0.44 _a_
		5.5	6.90 ± 0.37 _efg_	8.24 ± 0.44 _a_
	35	4.5	4.30 ± 0.23 _b_	8.10 ± 0.43 _a_
		5.0	4.30 ± 0.23 _b_	7.97 ± 0.42 _a_
		5.5	6.20 ± 0.33 _cd_	7.87 ± 0.42 _a_
4.34 ± 0.21	27	4.5	6.42 ± 0.31 _def_	8.21 ± 0.40 _a_
		5.0	7.08 ± 0.34 _fgh_	8.22 ± 0.40 _a_
		5.5	7.80 ± 0.38 _ij_	8.08 ± 0.39 _a_
	35	4.5	4.00 ± 0.19 _b_	8.01 ± 0.39 _a_
		5.0	5.74 ± 0.28 _cd_	8.11 ± 0.41 _a_
		5.5	7.06 ± 0.34 _fgh_	8.06 ± 0.37 _a_
5.34 ± 0.23	27	4.5	7.58 ± 0.33 _ghij_	8.10 ± 0.35 _a_
		5.0	8.20 ± 0.36 _jk_	8.12 ± 0.35 _a_
		5.5	8.24 ± 0.36 _jk_	8.14 ± 0.35 _a_
	35	4.5	6.00 ± 0.26 _cd_	7.96 ± 0.34 _a_
		5.0	7.42 ± 0.32 _ghi_	7.84 ± 0.34 _a_
		5.5	7.79 ± 0.34 _ij_	7.72 ± 0.33 _a_
6.34 ± 0.32	27	4.5	8.16 ± 0.41 _jk_	8.19 ± 0.41 _a_
		5.0	8.48 ± 0.43 _k_	8.20 ± 0.41 _a_
		5.5	8.62 ± 0.43 _k_	8.15 ± 0.41 _a_
	35	4.5	7.61 ± 0.38 _hij_	7.83 ± 0.39 _a_
		5.0	8.06 ± 0.41 _jk_	7.64 ± 0.38 _a_
		5.5	8.19 ± 0.41 _jk_	7.53 ± 0.38 _a_

* All sweet mash samples were inoculated with yeast 6.45 ± 0.32 log cfu/mL. a–j—Means with different lower-case letters for LAB and yeast are significantly different (three-way ANOVA, significance level 0.05).

**Table 2:** Results of chemical analysis of fermented mashes (data presented as Mean ± SD).

LAB Count in Sweet Mash log cfu/mL	Temperature (°C)	Initial pH	Fermented Mash *								
			Acetone ** (mg/L)	Isopropyl Alcohol (mg/L)	Acetaldehyde (mg/L)	Ethanol (g/L)	Residual Sugars (g/L)	Lactic Acid (g/L)	Acetic Acid (g/L)	Glycerol (g/L)	pH
Control (no inoculation with LAB)	27	4.5	0.62 ± 0.06 _ijklm_	0.46 ± 0.05 _ab_	66.54 ± 2.57 _mn_	53.51 ± 1.47 _ab_	1.43 ± 0.10 _no_	nd	0.15 ± 0.00 _ab_	3.02 ± 0.08 _a_	3.9 ± 0.1 _bc_
		5.0	0.51 ± 0.04 _b_	0.52 ± 0.05 _fgh_	54.68 ± 1.95 _k_	53.83 ± 1.18 _a_	1.24 ± 0.09 _ij_	nd	0.17 ± 0.00 _cd_	3.32 ± 0.07 _bc_	4.2 ± 0.1 _de_
		5.5	0.50 ± 0.05 _b_	0.59 ± 0.04 _k_	48.81 ± 1.29 _i_	58.86 ± 1.06 _bc_	1.23 ± 0.09 _hi_	nd	0.23 ± 0.00 _f_	3.71 ± 0.07 _fgh_	4.4 ± 0.1 _e_
	35	4.5	0.53 ± 0.05 _bcd_	0.55 ± 0.03 _hij_	69.85 ± 3.72 _no_	58.19 ± 1.02 _bc_	0.88 ± 0.07 _f_	nd	0.17 ± 0.00 _cd_	3.54 ± 0.06 _cdef_	4.0 ± 0.1 _cd_
		5.0	0.55 ± 0.02 _cde_	0.55 ± 0.04 _hij_	50.49 ± 1.62 _ij_	56.71 ± 1.27 _abc_	0.89 ± 0.08 _fg_	nd	0.20 ± 0.00 _e_	3.62 ± 0.08 _efg_	4.3 ± 0.1 _de_
		5.5	0.51 ± 0.04 _b_	0.66 ± 0.05 _m_	40.31 ± 1.38 _fg_	57.98 ± 1.31 _bc_	0.79 ± 0.07 _de_	nd	0.24 ± 0.01 _f_	3.99 ± 0.09 _ijk_	4.4 ± 0.1 _e_
3.34 ± 0.18	27	4.5	0.71 ± 0.03 _p_	0.48 ± 0.05 _abcd_	29.47 ± 1.60 _bc_	58.22 ± 1.93 _bc_	1.33 ± 0.09 _lm_	0.11 ± 0.00 _ab_	0.14 ± 0.00 _a_	3.32 ± 0.11 _bc_	4.0 ± 0.1 _cd_
		5.0	0.68 ± 0.06 _op_	0.46 ± 0.04 _abc_	23.61 ± 1.19 _a_	55.28 ± 1.59 _ab_	1.17 ± 0.08 _h_	0.15 ± 0.00 _b_	0.14 ± 0.00 _a_	3.56 ± 0.10 _cdef_	4.2 ± 0.1 _de_
		5.5	0.61 ± 0.05 _ghijk_	0.53 ± 0.02 _fghi_	30.81 ± 1.73 _c_	58.05 ± 2.00 _bc_	1.19 ± 0.09 _hi_	0.30 ± 0.01 _c_	0.23 ± 0.01 _f_	3.75 ± 0.13 _fgh_	4.5 ± 0.2 _e_
	35	4.5	0.64 ± 0.05 _klmno_	0.48 ± 0.03 _abcde_	31.16 ± 1.73 _c_	56.14 ± 1.93 _abc_	0.80 ± 0.07 _de_	0.08 ± 0.00 _ab_	0.16 ± 0.01 _bc_	3.39 ± 0.12 _bcd_	4.0 ± 0.1 _cd_
		5.0	0.56 ± 0.03 _def_	0.53 ± 0.03 _fghi_	43.62 ± 1.66 _gh_	55.35 ± 1.40 _ab_	0.90 ± 0.08 _fg_	0.10 ± 0.00 _ab_	0.24 ± 0.01 _f_	3.51 ± 0.09 _cdef_	4.3 ± 0.1 _de_
		5.5	0.50 ± 0.04 _b_	0.63 ± 0.04 _l_	40.02 ± 2.74 _ef_	55.55 ± 1.57 _ab_	0.74 ± 0.06 _cd_	0.28 ± 0.01 _c_	0.33 ± 0.01 _jk_	3.71 ± 0.11 _fgh_	4.5 ± 0.1 _e_
4.34 ± 0.21	27	4.5	0.71 ± 0.07 _p_	0.47 ± 0.02 _abc_	53.77 ± 2.20 _k_	57.04 ± 1.61 _abc_	1.38 ± 0.07 _mn_	0.20 ± 0.01 _bc_	0.14 ± 0.00 _ab_	3.23 ± 0.09 _ab_	4.0 ± 0.1 _cd_
		5.0	0.65 ± 0.06 _mno_	0.47 ± 0.04 _abc_	41.14 ± 1.80 _fg_	56.37 ± 1.63 _abc_	1.26 ± 0.08 _ijk_	0.52 ± 0.02 _d_	0.17 ± 0.00 _cd_	3.52 ± 0.10 _cdef_	4.3 ± 0.1 _de_
		5.5	0.59 ± 0.05 _efghi_	0.57 ± 0.02 _jk_	71.16 ± 2.44 _o_	54.68 ± 1.32 _ab_	1.16 ± 0.07 _h_	0.69 ± 0.02 _f_	0.27 ± 0.01 _gh_	3.62 ± 0.09 _defg_	4.4 ± 0.1 _e_
	35	4.5	0.53 ± 0.02 _bcd_	0.54 ± 0.02 _ghij_	63.74 ± 2.44 _m_	59.53 ± 1.60 _c_	0.95 ± 0.09 _g_	0.12 ± 0.00 _ab_	0.17 ± 0.00 _d_	3.72 ± 0.10 _fgh_	4.0 ± 0.1 _cd_
		5.0	0.56 ± 0.06 _def_	0.54 ± 0.05 _ghij_	36.35 ± 1.86 _de_	56.10 ± 1.86 _abc_	0.78 ± 0.08 _cde_	0.27 ± 0.01 _c_	0.26 ± 0.01 _g_	3.60 ± 0.12 _defg_	4.3 ± 0.1 _de_
		5.5	0.44 ± 0.04 _a_	0.67 ± 0.04 _m_	52.86 ± 1.54 _jk_	58.08 ± 1.16 _bc_	0.72 ± 0.06 _c_	0.71 ± 0.01 _f_	0.42 ± 0.01 _m_	4.00 ± 0.08 _ijk_	4.5 ± 0.1 _e_
5.34 ± 0.23	27	4.5	0.67 ± 0.03 _no_	0.49 ± 0.03 _bcdef_	35.43 ± 1.98 _d_	58.12 ± 2.09 _bc_	1.47 ± 0.11 _o_	0.66 ± 0.02 _ef_	0.16 ± 0.01 _cd_	3.38 ± 0.12 _bcde_	4.0 ± 0.1 _cd_
		5.0	0.62 ± 0.02 _hijklm_	0.50 ± 0.04 _cdef_	38.66 ± 1.46 _def_	57.58 ± 1.41 _bc_	1.32 ± 0.10 _klm_	1.44 ± 0.04 _g_	0.23 ± 0.01 _f_	3.74 ± 0.09 _fgh_	4.0 ± 0.1 _cd_
		5.5	0.58 ± 0.05 _efg_	0.51 ± 0.04 _dfg_	48.79 ± 1.09 _i_	54.13 ± 1.24 _ab_	1.31 ± 0.09 _jkl_	1.66 ± 0.04 _h_	0.29 ± 0.01 _i_	3.85 ± 0.09 _ghij_	4.1 ± 0.1 _cd_
	35	4.5	0.52 ± 0.05 _bc_	0.53 ± 0.03 _ghi_	30.87 ± 1.97 _bc_	58.72 ± 1.38 _bc_	0.84 ± 0.08 _ef_	0.56 ± 0.01 _de_	0.16 ± 0.00 _cd_	3.59 ± 0.08 _defg_	4.0 ± 0.1 _cd_
		5.0	0.59 ± 0.05 _fghij_	0.54 ± 0.04 _ghij_	27.15 ± 1.66 _b_	57.94 ± 2.11 _bc_	0.61 ± 0.07 _b_	1.56 ± 0.06 _h_	0.32 ± 0.01 _jk_	4.05 ± 0.15 _jk_	4.0 ± 0.1 _cd_
		5.5	0.63 ± 0.06 _jklmn_	0.52 ± 0.07 _efgh_	53.41 ± 1.08 _jk_	58.48 ± 1.53 _bc_	0.50 ± 0.07 _a_	2.73 ± 0.07 _j_	0.38 ± 0.01 _l_	4.33 ± 0.11 _l_	4.0 ± 0.1 _cd_
6.34 ± 0.32	27	4.5	0.59 ± 0.04 _efghi_	0.47 ± 0.02 _abc_	45.78 ± 2.02 _h_	57.01 ± 1.73 _bc_	1.33 ± 0.10 _lm_	1.83 ± 0.06 _i_	0.23 ± 0.01 _f_	3.73 ± 0.11 _fghi_	3.7 ± 0.1 _a_
		5.0	0.62 ± 0.06 _ijklm_	0.47 ± 0.03 _abc_	97.06 ± 5.82 _p_	56.59 ± 2.52 _abc_	1.40 ± 0.09 _n_	3.05 ± 0.14 _k_	0.28 ± 0.01 _hi_	4.90 ± 0.22 _m_	3.7 ± 0.2 _a_
		5.5	0.65 ± 0.06 _lmno_	0.52 ± 0.06 _defgh_	120.68 ± 5.77 _s_	56.07 ± 1.33 _abc_	1.30 ± 0.09 _jkl_	4.46 ± 0.11 _m_	0.33 ± 0.01 _k_	3.80 ± 0.09 _ghij_	3.7 ± 0.1 _a_
	35	4.5	0.58 ± 0.05 _efghi_	0.53 ± 0.06 _ghi_	30.55 ± 1.69 _bc_	58.47 ± 1.98 _bc_	0.77 ± 0.07 _cd_	1.80 ± 0.06 _i_	0.26 ± 0.01 _g_	3.93 ± 0.13 _hij_	3.8 ± 0.1 _ab_
		5.0	0.59 ± 0.04 _efghi_	0.46 ± 0.05 _a_	58.59 ± 3.17 _l_	59.03 ± 1.52 _bc_	0.53 ± 0.08 _a_	3.70 ± 0.10 _l_	0.32 ± 0.01 _j_	4.21 ± 0.11 _kl_	3.8 ± 0.1 _ab_
		5.5	0.61 ± 0.05 _ghijkl_	0.45 ± 0.02 _a_	114.03 ± 6.04 _r_	53.35 ± 1.43 _a_	0.48 ± 0.06 _a_	4.64 ± 0.12 _n_	0.37 ± 0.01 _l_	3.90 ± 0.10 _hij_	3.8 ± 0.1 _ab_

* Total soluble solids and total sugar content in sweet mashes 150.1 ± 9.2 g/kg and 129.2 ± 8.3 g glucose/L; ** Initial acetone content 1.38 mg/L; a–s—Means with different lower-case letters in columns are significantly different (three-way ANOVA, significance level 0.05).

**Table 3:** Results of three-way ANOVA.

Factors	Fermented Mash										
	Acetone	Isopropyl Alcohol	Acetaldehyde	Residual Sugars	Lactic Acid	Acetic Acid	Glycerol	Ethanol	pH	LAB	Yeast
LABC	***	***	***	***	***	***	***	ns	***	***	ns
T	***	***	***	***	***	***	***	*	ns	***	**
IpH	***	***	***	***	***	***	***	*	***	***	ns
LABC * T	***	***	***	***	***	***	***	*	ns	***	ns
LABC * IpH	***	***	***	***	***	***	***	**	***	***	ns
T * IpH	***	ns	***	*	***	***	***	ns	ns	**	ns
LABC * T * IpH	***	***	***	***	***	***	***	*	ns	*	ns

LABC—Lactic acid bacteria count in sweet mash; T—Temperature; IpH—Initial pH of sweet mash; *** *p* < 0.001, ** *p* < 0.01, * *p* < 0.05, ns—Not significant.
